# Prognostic Value of the CALLY Index in Predicting All-Cause Mortality After Transcatheter Aortic Valve Implantation: A Two-Year Follow-Up Study

**DOI:** 10.3390/medicina62040755

**Published:** 2026-04-15

**Authors:** Zeynep Esra Güner, İsmail Balaban, Mustafa Ferhat Keten, Rıdvan Bolataslan, Ravza Betül Akbaş, Seda Tanyeri Üzel, Regayip Zehir, Elnur Alizade

**Affiliations:** 1 Department of Cardiology, Uzunköprü State Hospital, Edirne 22000, Turkey; 2 Department of Cardiology, Kartal Koşuyolu High Specialization Training and Research Hospital, Istanbul 34846, Turkey; ismail.balaban2@saglik.gov.tr (İ.B.); mustafa.keten1@saglik.gov.tr (M.F.K.); ridvan.bolataslan@saglik.gov.tr (R.B.); seda.tanyeri@saglik.gov.tr (S.T.Ü.); regayip.zehir@sbu.edu.tr (R.Z.); elnur.alizade@sbu.edu.tr (E.A.); 3 Deparment of Cardiology, Bitlis Tatvan State Hospital, Bitlis 13200, Turkey; ravzabetul.akbas@saglik.gov.tr

**Keywords:** transcatheter aortic valve implantation, CALLY index, all-cause mortality, aortic stenosis, inflammatory biomarkers

## Abstract

*Background and Objectives*: This study investigated the prognostic value of the C-reactive protein–albumin–lymphocyte (CALLY) index in predicting all-cause mortality among patients undergoing transcatheter aortic valve implantation (TAVI) for severe aortic stenosis. *Materials and methods*: This retrospective single-center study included 303 patients who underwent TAVI. The CALLY index and other established prognostic scores were calculated at baseline. Patients were followed for a median of 21 months. The primary endpoint was all-cause mortality. *Results*: A total of 60 patients (19.8%) died during follow-up. The CALLY index demonstrated the highest predictive performance for all-cause mortality, with an AUC of 0.698 (95% CI: 0.628–0.768, *p* < 0.001). In multivariate Cox regression, a low CALLY index remained an independent predictor of mortality (HR: 3.80, 95% CI: 2.03–7.11, *p* < 0.001), along with reduced LVEF, chronic kidney disease, and diabetes mellitus. Kaplan–Meier analysis further confirmed markedly worse survival in the high-risk group (log-rank *p* < 0.001). *Conclusions*: The CALLY index was independently associated with mortality after TAVI and may represent a complementary biomarker for risk stratification in this population.

## 1. Introduction

Aortic stenosis (AS) is a common valvular heart disease among elderly individuals and is associated with high morbidity and mortality if left untreated [1]. In recent years, TAVI has emerged as a safe and effective alternative to surgical aortic valve replacement (SAVR), particularly in older patients with a high surgical risk [2]. However, despite procedural advancements, post-TAVI mortality and complication rates remain clinically significant owing to the advanced age, comorbidities and frailty of this patient population. Therefore, identifying novel biomarkers and risk assessment models that can more accurately predict the prognosis in TAVI patients is of great clinical importance [3].

Inflammation and nutritional status, as fundamental biological processes in the pathogenesis and progression of aortic stenosis, play key roles in disease advancement and significantly influence clinical outcomes after TAVI. Therefore, biomarkers integrating inflammatory and nutritional status may provide additional prognostic insight beyond traditional clinical variables [4]. Furthermore, the interaction between malnutrition and systemic inflammation disrupts tissue repair mechanisms and contributes to the progressive stiffening of the valve leaflets [5]. These pathophysiological mechanisms play a critical role not only in the initiation of the disease but also in determining its rate of progression and clinical outcomes.

Although increasing evidence indicates that inflammation and malnutrition are associated with adverse cardiovascular outcomes, their combined impact on patients with aortic stenosis remains insufficiently defined. In particular, data on the nutritional status of older adults undergoing aortic valve replacement and its clinical relevance to postprocedural prognosis are limited. Considering the underlying inflammatory and nutrition-related mechanisms of the disease, the CALLY index, which provides a comprehensive reflection of the interaction between these two systems, is thought to offer additional prognostic value for risk assessment in patients undergoing TAVI.

The CALLY index, calculated using the formula (albumin × lymphocyte/CRP), is an innovative composite biomarker that integrates systemic inflammation, immune system activity, and nutritional status into a single measure. In cardiovascular diseases, particularly acute coronary syndrome (ACS) and ST-segment elevation myocardial infarction (STEMI), lower CALLY scores have been reported to be significantly associated with increased mortality and adverse events [6,7]. Moreover, studies in oncology have confirmed the CALLY index as an independent prognostic indicator across various malignancies [8,9,10].

Previous studies have demonstrated that inflammation and nutrition based indicators such as the Naples Prognostic Score (NPS), Prognostic Nutritional Index (PNI), Controlling Nutritional Status (CONUT) score, Systemic Immune-Inflammation Index (SII) and Geriatric Nutritional Risk Index (GNRI) are significantly associated with mortality and adverse cardiovascular events following TAVI [11,12,13,14,15]. However, the role of the CALLY index in patients undergoing TAVI remains unclear. This gap highlights the need for contemporary, large-scale, and prospective studies that could improve TAVI-specific risk stratification, incorporate inflammatory and nutritional mechanisms into clinical practice, and enable a more accurate prediction of patient prognosis.

In light of this evidence, the present study aimed to evaluate the prognostic significance of the CALLY index in patients with aortic stenosis undergoing TAVI and examine its association with all-cause mortality.

## 2. Material and Methods

### 2.1. Study Population

This was a single-center, retrospective cohort study. Between January 2018 and December 2023, consecutive patients diagnosed with severe symptomatic aortic stenosis (AS) who underwent transcatheter aortic valve implantation (TAVI) were evaluated in this study. Approximately 440 patients were initially screened.

The inclusion criteria were as follows: age ≥ 18 years; echocardiographically confirmed diagnosis of severe AS (mean pressure gradient > 40 mmHg, peak jet velocity > 4.0 m/s, or aortic valve area < 1.0 cm^2^); successful transfemoral TAVI procedure; and availability of complete clinical, laboratory, and echocardiographic data before and after the intervention.

The exclusion criteria were as follows: multivalvular intervention (patients undergoing surgical or percutaneous treatment of more than one valve; *n* = 28), chronic hemodialysis or peritoneal dialysis therapy (*n* = 18), severe infection, sepsis, or hemodynamic shock at presentation (*n* = 16), intraprocedural mortality (*n* = 18), chronic rheumatologic or autoimmune diseases (e.g., systemic lupus erythematosus, rheumatoid arthritis, vasculitis; *n* = 21), and ongoing immunosuppressive therapy (e.g., corticosteroids, biologic agents, or cytotoxic drugs; *n* = 12).

After applying the exclusion criteria, 303 patients were deemed eligible and included in the final analysis. A multidisciplinary Heart Team preoperatively evaluated all patients to confirm the indication for TAVI.

All data were retrieved from the institutional electronic medical record system and validated for completeness and accuracy through cross-verification with the national health and death registries via the e-Nabız digital health platform, which serves as Turkey’s National Personal Health Record System [16].

Ethical approval for this study was obtained from by the Clinical Research Ethics Committee of Kartal Koşuyolu High Specialization Training and Research Hospital (Approval Number: 2025/16/1243; date: 30 September 2025). This study was conducted in accordance with the ethical principles outlined in the Declaration of Helsinki.

### 2.2. Preprocedural Assessment and Procedural Technique

Patients diagnosed with symptomatic and severe aortic stenosis who were evaluated as candidates for TAVI underwent comprehensive preprocedural assessment. This evaluation included a detailed clinical examination, routine laboratory tests, coronary angiography, transthoracic echocardiography (TTE) to confirm the severity of AS, and contrast-enhanced computed tomography angiography (CTA) to assess the anatomy of the aortic root, annulus, and vascular access routes. All collected data were reviewed by a multidisciplinary Heart Team consisting of interventional cardiologists, cardiac surgeons, anesthesiologists, and radiologists to determine the patient’s eligibility and optimal procedural strategy.

For eligible patients, the procedure was performed electively via a percutaneous transfemoral approach under deep sedation or general anesthesia, depending on the patient’s clinical condition and institutional protocol. In most cases, vascular access was achieved percutaneously via the right femoral artery. A temporary pacemaker was inserted via the femoral route to enable rapid ventricular pacing and prevent potential atrioventricular conduction disturbances during the procedure.

Anticoagulation was achieved with unfractionated heparin. Two ProGlide (Abbott Vascular, Santa Clara, CA, USA) vascular closure devices were routinely pre-implanted to facilitate percutaneous closure at the end of the procedure. Based on anatomical and clinical suitability determined by CTA measurements, either self-expanding bioprosthetic valves (e.g., CoreValve, Evolut R/Pro, Portico, and ACURATE neo) or balloon-expandable valves (e.g., Edwards SAPIEN XT, S3, and SAPIEN 3 Ultra) were implanted. Following valve deployment, control angiography was performed to assess device positioning and exclude aortic regurgitation, paravalvular leak, dissection, or other vascular complications.

**Figure 1:**
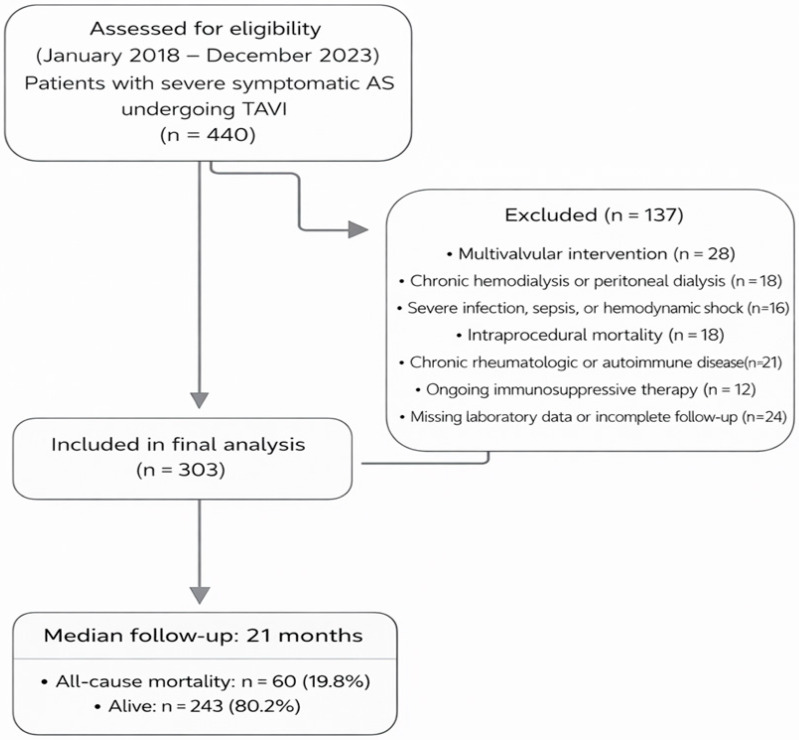
Study Flowchart.

After the procedure, transthoracic echocardiography was performed in all patients to evaluate prosthetic valve function and detect any subclinical complications, such as pericardial effusion or paravalvular leak. Postprocedural medical therapy was planned in accordance with the current European Society of Cardiology (ESC) and European Association for Cardio-Thoracic Surgery (EACTS) guidelines for the management of valvular heart disease. The detailed patient selection process is illustrated in Figure 1.

### 2.3. Follow Up and Study Outcome

Patients were clinically followed up for a median duration of 21 months through scheduled outpatient visits, telephone interviews, and review of the national electronic health record system. None of the patients were lost to follow-up. The primary endpoint of the study was defined as all-cause mortality. Mortality data were obtained from the institutional medical records and verified using the National Death Notification System. All procedural and postprocedural clinical events were defined and classified according to the standardized criteria of the Valve Academic Research Consortium-3(VARC-3) [17].

### 2.4. Statistical Analysis

All statistical analyses were performed using SPSS (version 27), Jamovi (version 2.7.6), and Python (version 3.13) for advanced computation and visualization.

Normally distributed continuous variables are presented as mean ± standard deviation, whereas non-normally distributed variables are presented as median (minimum–maximum). Categorical variables are expressed as frequencies and percentages. Comparisons between two groups were performed using the independent samples *t*-test or Mann–Whitney U test for continuous variables and the chi-square test or Fisher’s exact test for categorical variables, as appropriate. ROC curve analysis was performed to assess the predictive accuracy of continuous variables and to identify optimal cutoff thresholds. The optimal cut-off value for the CALLY index was determined using the Youden index, which identifies the point maximizing the sum of sensitivity and specificity. Survival analysis was conducted using the Kaplan–Meier method, and survival curves were compared using the log-rank test. Univariate Cox proportional hazards regression analysis was initially performed to evaluate predictors of all-cause mortality. Variables with a *p*-value < 0.10 in univariate analysis were subsequently entered into a multivariate Cox regression model to identify independent predictors. Hazard ratios (HRs) with 95% confidence intervals (CIs) are reported.

A two-sided *p*-value < 0.05 was considered statistically significant for all analyses.

### 2.5. Laboratory Measurements and Calculation of the CALLY Score

Venous blood samples were collected after overnight fasting within 24 h before TAVI. Routine biochemical and hematological parameters, including albumin, CRP, cholesterol, neutrophil, lymphocyte, and monocyte counts, were measured in the hospital’s central laboratory using standardized automated analyzers. All laboratory personnel were blinded to the clinical data and outcomes.

The CALLY index was calculated as:

Albumin (g/L) × lymphocytes (10^9^/L) ÷ CRP (mg/L)

The Prognostic Nutritional Index (PNI) was calculated as:

10 × albumin (g/dL) + 0.005 × lymphocyte count (/mm^3^)

The CONUT score was calculated from serum albumin, total lymphocyte count, and total cholesterol levels using the standard scoring system.

The Naples Prognostic Score (NPS) was determined based on serum albumin, total cholesterol, the NLR, and the LMR, with each parameter assigned 1 point according to validated cutoff values.

The Geriatric Nutritional Risk Index (GNRI) was calculated using the following formula:

GNRI = (1.489 × albumin [g/L]) + (41.7 × current body weight/ideal body weight)

The ideal body weight was calculated using the formula 22 × height^2^ (m^2^). When the ratio of current body weight to ideal body weight was greater than 1, this value was set to 1.

All indices were calculated using previously validated formulas, as described in the original studies.

## 3. Results

### 3.1. Baseline Characteristics and Medical Treatment

Baseline characteristics according to survival status are presented in Table 1. During the follow-up period, 60 patients (19.8%) died. The mean age was similar between survivors and non-survivors (78.0 ± 6.2 vs. 79.1 ± 6.6 years, *p* = 0.240), and the sex distribution did not differ significantly between the groups (*p* = 0.790). Non-survivors had significantly lower left ventricular ejection fraction than survivors (44.7 ± 14.4% vs. 60.3 ± 8.6%, *p* < 0.001). The prevalence of atrial fibrillation (45.0% vs. 20.2%, *p* < 0.001), hypertension (86.7% vs. 69.5%, *p* = 0.008), diabetes mellitus (56.7% vs. 28.8%, *p* < 0.001), chronic kidney disease (38.3% vs. 8.2%, *p* < 0.001), smoking history (30.0% vs. 15.2%, *p* = 0.008), previous coronary artery bypass grafting (30.5% vs. 16.3%, *p* = 0.012), and previous valve surgery (11.9% vs. 2.1%, *p* = 0.001) was significantly higher among patients who died. Other clinical variables, including hyperlipidemia, cerebrovascular disease, chronic obstructive pulmonary disease, peripheral artery disease, previous percutaneous coronary intervention, coronary artery disease, and anemia, were not significantly different between survivors and non-survivors (all *p* > 0.05).

#### CALLY Risk Stratification

The baseline characteristics according to the CALLY risk stratification are presented in Table 2. Patients in the high-risk CALLY group (≤1.11) were significantly older and had lower ejection fractions than those in the low-risk group (>1.11).

The high-risk group exhibited markedly higher two-year mortality (35.5% vs. 9.3%, *p* < 0.001). Comorbid conditions, including hypertension, diabetes mellitus, atrial fibrillation, chronic kidney disease, and anemia, were significantly more prevalent in the high-risk group.

The shorter median follow-up duration observed in the high-risk group reflects earlier mortality rather than a differential follow-up intensity.

### 3.2. Medication Use

Medication use according to CALLY risk status is summarized in Table 3. Most pharmacological treatments were similarly distributed between groups. However, diuretic use was significantly more frequent in the low-risk group (*p* = 0.009). No significant differences were observed in anticoagulant, antiplatelet, beta-blocker, ACEi/ARB, MRA, or statin therapy.

### 3.3. Laboratory Parameters and Prognostic Score Values

Laboratory parameters and prognostic score values stratified by CALLY risk status are presented in Table 4.

Among hematological parameters, the high-risk CALLY group demonstrated significantly lower lymphocyte, hemoglobin, WBC, and platelet counts, along with higher CRP levels and inflammatory indices (NLR, PLR), consistent with heightened systemic inflammation and impaired immune–nutritional reserve.

Biochemical parameters further supported this profile. High-risk patients had significantly lower albumin and PNI values and higher HbA1c and creatinine levels. These findings reinforce the biological plausibility of the CALLY index as a composite marker integrating inflammatory burden, nutritional status, and renal dysfunction.

### 3.4. ROC Analysis, Cox Regression Models and Survival Outcomes

**Figure 2:**
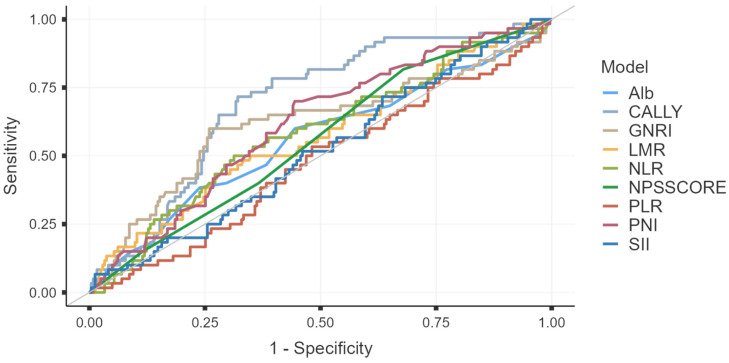
Receiver operating characteristic (ROC) curves of nutritional and inflammatory indices for predicting two-year all-cause mortality. Abbreviations: CALLY, CRP–Albumin–Lymphocyte Index; CONUT, Controlling Nutritional Status; LMR, Lymphocyte-to-Monocyte Ratio; NLR, Neutrophil-to-Lymphocyte Ratio; PLR, Platelet-to-Lymphocyte Ratio; PNI, Prognostic Nutritional Index; SII, Systemic Inflammatory Index; NPSSCORE: Naples Score; Alb: Albumin; GNRI, Geriatric Nutritional Risk Index.

The discriminative performance of eight inflammatory and nutritional biomarkers (CALLY index, PNI, CONUT score, NAPLES score, NLR, PLR, LMR, SII, and GNRI) for predicting two-year all-cause mortality was evaluated using ROC curve analysis (Table 5). Among the evaluated indices, the CALLY index achieved the highest area under the curve (AUC), although its overall discriminatory ability remained moderate (AUC: 0.698; 95% CI: 0.628–0.768; *p* < 0.001). Albumin and GNRI demonstrated comparable but slightly lower predictive performance. In contrast, NLR, PLR, LMR, SII, and other composite scores showed weaker discriminative capacity, with ROC curves approaching the diagonal reference line, indicating limited predictive accuracy (Figure 2). The optimal cutoff value for the CALLY index was determined using the Youden index (≤1.10), which provided a sensitivity of 71.67% and specificity of 67.90%.

**Figure 3:**
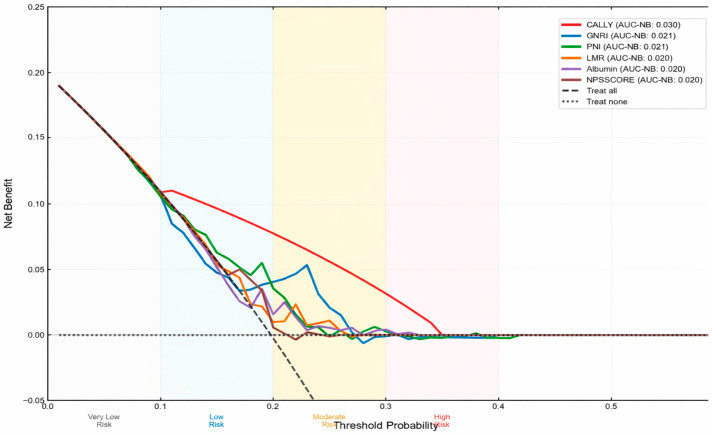
Decision Curve Analysis—Top 6 Performing Indices.

Decision curve analysis showed that the CALLY index yielded the highest net clinical benefit across a range of clinically relevant threshold probabilities (Figure 3).

**Figure 4:**
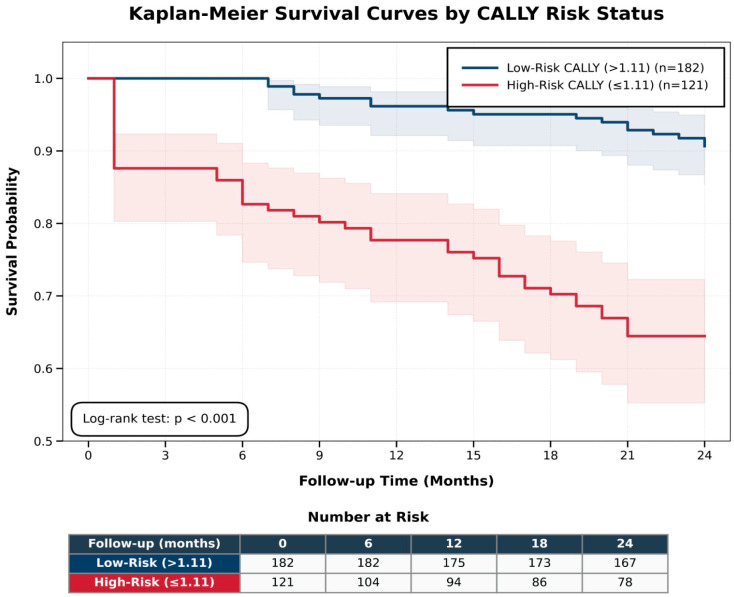
Kaplan–Meier survival curves stratified by the CALLY index cutoff.

According to the Cox regression analysis presented in Table 6 and Figure 4 a low CALLY index (≤1.10) was independently associated with all-cause mortality (HR 3.80; 95% CI 2.03–7.11; *p* < 0.001), along with reduced LVEF, chronic kidney disease and diabetes mellitus. Kaplan–Meier survival curves stratified by the CALLY index demonstrated significantly lower survival rates in the high-risk group, and this difference between groups was statistically significant (log-rank *p* < 0.001).

## 4. Discussion

In this retrospective cohort study, we demonstrated that the CALLY index, an integrated biomarker reflecting systemic inflammation, nutritional status, and immune competence, is significantly associated with two-year all-cause mortality after TAVI. A low CALLY index was strongly linked to a higher mortality risk, and this association remained robust after adjusting for established prognostic markers, such as reduced LVEF, chronic kidney disease, and diabetes mellitus. It should also be acknowledged that patients in the high-risk CALLY group were older than those in the low-risk group. Because age is a well-established prognostic factor in TAVI populations, propensity score adjustment for age was performed to address this potential imbalance. Importantly, the association between the CALLY index and mortality remained significant after this adjustment, suggesting that the prognostic value of the CALLY index cannot be explained solely by age differences between the groups (Supplementary Figure S1). Although the discriminatory ability of the CALLY index was modest, it performed relatively better than the other evaluated biomarkers (AUC: 0.698; 95% CI: 0.628–0.768), and Kaplan–Meier curves were consistent with the observed association between lower CALLY values and higher mortality risk.

The prognostic relevance of inflammation and nutrition based biomarkers in cardiovascular disease has gained increasing attention in recent years [18]. In the context of aortic stenosis, chronic low-grade inflammation has been implicated in the pathobiology of valvular calcification, endothelial dysfunction, and progressive myocardial remodeling [19]. However, it is important to distinguish this long-term inflammatory contribution to valve degeneration from the systemic inflammatory milieu that may influence clinical outcomes after TAVI.

In the periprocedural and postprocedural settings, systemic inflammation reflects a complex interplay between the baseline inflammatory burden, procedural stress, endothelial activation, and immune response. Sinning et al. first introduced the concept of systemic inflammatory response syndrome (SIRS) following TAVI, demonstrating that an exaggerated inflammatory response after the intervention is associated with increased short- and mid-term mortality [20]. In this context, inflammation is not merely a driver of valvular disease progression but a determinant of post-procedural vulnerability and adverse outcomes.

Furthermore, malnutrition and diminished immune reserve may impair tissue recovery, delay functional improvement, and predispose patients to infection and non-cardiovascular complications. Prior studies have shown that nutritional risk indices influenced by inflammatory status such as PNI, GNRI, NPS, and CONUT as well as hematologic inflammation-based markers including SII, NLR, PLR, and LMR, are consistently associated with mortality and major cardiovascular events after TAVR [21,22,23]. Similarly, other composite inflammation-nutritional indices, such as the advanced lung cancer inflammation index (ALI), have also been shown to predict adverse outcomes in cardiovascular conditions, including ST-segment elevation myocardial infarction [24]. These findings support the concept that integrated biomarkers capturing both inflammatory burden and nutritional reserve may provide incremental prognostic information beyond traditional clinical risk scores.

CRP reflects elevated systemic inflammation, whereas albumin serves as a negative acute-phase reactant, indicating both nutritional status and overall inflammatory burden. Multiple studies have shown that increased CRP levels are consistently associated with all-cause mortality after TAVI [25]. Similarly, data from the multicenter OCEAN-TAVI registry and other cohorts have demonstrated that low serum albumin levels significantly contribute to mortality risk in this population [26,27]. Lymphocyte count, a marker of immune reserve, has also been linked to immunosuppression and adverse clinical outcomes. Studies showing the prognostic importance of preprocedural lymphocyte levels and lymphocyte-based inflammatory markers such as NLR further support the concept that reduced immune reserve and heightened inflammation are key determinants of TAVI outcomes [28].

Unlike biomarkers that primarily reflect a single biological pathway, such as hematologic inflammation markers or nutrition-oriented indices that are indirectly influenced by inflammatory status, the CALLY index integrates inflammatory burden (CRP), nutritional reserve (albumin), and immune competence (lymphocyte count) into a unified composite measure. This multidimensional structure may partly explain the slightly better discriminative performance in this study.

Although it is not intended to function as a standalone diagnostic tool, the CALLY index offers meaningful complementary value. Its low cost, ease of calculation, and universal availability make it an attractive additive biomarker that can enhance existing prognostic models by providing biologically integrated insights into patient risk. From a clinical perspective, lower CALLY values may help identify individuals who require closer surveillance and more intensive post-procedural management, whereas higher CALLY scores may signify lower-risk patients who might benefit from less intensive follow-up, thereby supporting a more efficient use of healthcare resources.

Additionally, it should be acknowledged that established surgical risk scores, such as the STS score or EuroSCORE II, as well as formal frailty assessments, were not systematically available in our retrospective dataset and therefore could not be incorporated into the multivariate adjustment. Given the well-established prognostic importance of frailty and global clinical risk burden in TAVI populations, it is possible that part of the prognostic signal captured by the CALLY index reflects broader patient vulnerability rather than a purely distinct biological pathway. Consequently, residual confounding cannot be completely excluded, and our findings should be interpreted in this context. Furthermore, the components of the CALLY index were measured at a single preprocedural time point, within 24 h before TAVI. Because CRP is a dynamic inflammatory marker and albumin levels may be influenced by hydration status or acute-phase responses, a single measurement may partially reflect transient inflammatory activity rather than a stable biological phenotype. Nevertheless, this approach reflects routine clinical practice and may capture the integrated inflammatory-nutritional status of the patient at the time of the procedure. Future prospective studies incorporating serial biomarker measurements may help clarify whether longitudinal changes in the CALLY index provide additional prognostic value.

### Limitations

This study has several limitations. First, the retrospective and single-center design inherently carries a risk of selection bias and may restrict the external generalizability of the findings. Second, because biomarkers such as CRP, albumin, and lymphocyte count were measured only at baseline, temporal changes in inflammatory or nutritional status could not be evaluated. Additionally, residual confounding due to unmeasured factors such as frailty assessments or detailed nutritional evaluations cannot be completely excluded. Therefore, these results require confirmation in larger, prospective, and multicenter cohorts.

## 5. Conclusions

This study suggests that the CALLY index is associated with all-cause mortality after TAVI. Although its overall discriminative ability was limited, its relatively favorable performance compared with other immune-inflammatory biomarkers supports the potential use of the CALLY index as a practical, low-cost, and complementary tool for post-TAVI risk stratification.

## Figures and Tables

**Table 1:** Baseline Characteristics of Patients According to Survival Status.

Characteristic	Total Cohort (*n* = 303)	Survivors (*n* = 243)	Non Survivors (*n* = 60)	*p*-Value
Age, years	78.2 ± 6.3	78.0 ± 6.2	79.1 ± 6.6	0.240
Sex				0.790
Female	162 (53.5)	129 (53.1)	33 (55.0)	
Male	141 (46.5)	114 (46.9)	27 (45.0)	
Ejection fraction, %	57.2 ± 11.8	60.3 ± 8.6	44.7 ± 14.4	<0.001
Atrial fibrillation	76 (25.1)	49 (20.2)	27 (45.0)	<0.001
Hypertension	221 (72.9)	169 (69.5)	52 (86.7)	0.008
Diabetes mellitus	104 (34.3)	70 (28.8)	34 (56.7)	<0.001
Hyperlipidemia	87 (28.9)	68 (28.1)	19 (32.2)	0.533
Chronic kidney disease	43 (14.2)	20 (8.2)	23 (38.3)	<0.001
Smoking history	55 (18.2)	37 (15.2)	18 (30.0)	0.008
Cerebrovascular disease	18 (6.0)	14 (5.8)	4 (6.7)	0.796
COPD	41 (13.6)	33 (13.6)	8 (13.3)	0.951
Peripheral artery disease	8 (2.7)	6 (2.5)	2 (3.4)	0.708
Coronary artery disease	145 (47.9)	110 (45.3)	35 (58.3)	0.070
Previous CABG	57 (19.1)	39 (16.3)	18 (30.5)	0.012
Previous valve surgery	12 (4.0)	5 (2.1)	7 (11.9)	0.001
Previous PCI	66 (22.4)	49 (20.8)	17 (28.8)	0.184
Anemia	122 (40.4)	92 (38.0)	30 (50.0)	0.090

Abbreviations: COPD, chronic obstructive pulmonary disease; CABG, coronary artery bypass grafting; PCI, percutaneous coronary intervention.

**Table 2:** Baseline Demographic and Clinical Characteristics Stratified by CALLY Risk Stratification.

Characteristic	Total (*n* = 303)	Low Risk CALLY (>1.11)	High-Risk CALLY (≤1.11)	*p*-Value
Age, years	78 (63–92)	77 (63–91)	80 (63–92)	<0.001
Sex, *n* (%)				0.311
Female	162 (53.5)	93 (51.1)	69 (57.0)	
Male	141 (46.5)	89 (48.9)	52 (43.0)	
Height, m	1.62 (1.37–1.88)	1.62 (1.37–1.88)	1.62 (1.45–1.80)	0.694
Weight, kg	74 (51–140)	74 (51–140)	75 (52–120)	0.601
Body mass index, kg/m^2^	28.43 (19.47–49.60)	28.33 (19.96–49.60)	28.57 (19.47–42.22)	0.631
Ejection fraction, %	57 (20–65)	58 (20–65)	55 (20–65)	0.034
Two-year mortality, *n* (%)	60 (19.8)	17 (9.3)	43 (35.5)	<0.001
Follow-up time, months	21 (1–24)	23 (7–24)	19 (1–24)	<0.001
Comorbidities, *n* (%)				
Atrial fibrillation	76 (25.1)	38 (20.9)	38 (31.4)	0.038
Hypertension	221 (72.9)	120 (65.9)	101 (83.5)	0.001
Diabetes mellitus	104 (34.3)	51 (28.0)	53 (43.8)	0.005
Hyperlipidemia	87 (28.9)	54 (29.8)	33 (27.5)	0.662
Chronic kidney disease	43 (14.2)	14 (7.7)	29 (24.0)	<0.001
Smoking	55 (18.2)	30 (16.5)	25 (20.7)	0.355
Cerebrovascular disease	18 (6.0)	10 (5.5)	8 (6.7)	0.674
COPD	41 (13.6)	22 (12.2)	19 (15.7)	0.378
Peripheral arterial disease	8 (2.7)	2 (1.1)	6 (5.0)	0.063
Cardiac interventions, *n* (%)				
CABG	57 (19.1)	32 (17.7)	25 (21.2)	0.451
Valve surgery	12 (4.0)	4 (2.2)	8 (6.8)	0.069
PCI	66 (22.4)	32 (17.9)	34 (29.3)	0.021
Coronary artery disease, *n* (%)	145 (47.9)	80 (44.0)	65 (53.7)	0.096
Anemia, *n* (%)	122 (40.4)	54 (29.8)	68 (56.2)	<0.001

Abbreviations: Continuous variables are presented as median (minimum–maximum), and categorical variables as *n* (%). Percentages represent column percentages within each CALLY risk group. Continuous variables were compared using the Mann–Whitney U test. Categorical variables were compared using Pearson’s chi-square test or Fisher’s exact test when expected cell counts were less than 5. A two-sided *p*-value < 0.05 was considered statistically significant.

**Table 3:** Medication Use of Patients.

Medication, *n* (%)	Low Risk CALLY (>1.11) (*n* = 175)	High Risk CALLY (≤1.11) (*n* = 119)	*p*-Value
Warfarin	13 (7.4%)	13 (10.9%)	0.300
NOAC	33 (18.9%)	22 (18.5%)	0.936
ASA	103 (59.2%)	63 (53.4%)	0.326
Clopidogrel	68 (38.9%)	51 (42.9%)	0.493
Beta-blocker	123 (69.9%)	90 (75.6%)	0.280
ACEi/ARB	104 (59.1%)	75 (63.0%)	0.497
MRA	30 (17.2%)	13 (11.0%)	0.141
Diuretic	117 (66.5%)	61 (51.3%)	0.009
Statin	94 (53.4%)	61 (51.3%)	0.717

Abbreviations: Data are presented as *n* (%). Percentages represent the proportion of patients using the medication within each CALLY risk group. NOAC: Non-vitamin K antagonist oral anticoagulant; ASA: Acetylsalicylic acid; ACEi/ARB: Angiotensin-converting enzyme inhibitor/Angiotensin receptor blocker; MRA: Mineralocorticoid receptor antagonist.

**Table 4:** Laboratory Parameters and Prognostic Score Values of the Study Population.

Parameter	Low CALLY Risk (*n* = 175)	High CALLY Risk (*n* = 119)	
	Mean ± SD (Min–Max)	Mean ± SD (Min–Max)	*p*
**Hematological Parameters**			
Neutrophil (10^3^/µL)	5.03 ± 1.78 (2.00–11.54)	4.89 ± 2.11 (1.80–15.14)	0.522
Lymphocyte (10^3^/µL)	1.97 ± 0.62 (0.86–3.80)	1.24 ± 0.43 (0.25–2.90)	<0.001
Monocyte (10^3^/µL)	0.65 ± 0.22 (0.15–1.70)	0.57 ± 0.25 (0.10–1.79)	0.007
Platelet (10^3^/µL)	235.7 ± 76.6 (78–542)	216.9 ± 74.0 (64–443)	0.036
WBC (10^3^/µL)	7.72 ± 1.94 (3.91–13.99)	6.64 ± 2.27 (3.00–17.88)	<0.001
Hemoglobin (g/dL)	12.58 ± 1.65 (8.00–16.80)	11.52 ± 1.47 (7.50–15.70)	<0.001
CRP (mg/L)	4.42 ± 1.62 (0.90–9.60)	6.28 ± 1.47 (3.00–9.00)	<0.001
NLR	2.78 ± 1.35 (0.86–8.93)	4.67 ± 3.98 (1.00–36.68)	<0.001
LMR	3.47 ± 1.92 (0.86–18.00)	2.55 ± 1.46 (0.53–11.54)	<0.001
PLR	128.2 ± 56.3 (0.00–361.0)	201.4 ± 126.6 (0.00–1048.0)	<0.001
**Biochemical Parameters**			
Albumin (g/L)	40.8 ± 3.5 (30.0–59.0)	38.1 ± 4.9 (24.0–48.0)	<0.001
PNI	60.5 ± 6.9 (42.3–81.0)	50.5 ± 6.4 (31.7–62.0)	<0.001
Cholesterol (mg/dL)	194.7 ± 46.0 (108–333)	185.1 ± 48.7 (79–351)	0.081
HbA1c (%)	6.3 ± 0.7 (5.1–9.1)	6.7 ± 1.0 (5.1–9.5)	<0.001
Creatinine (mg/dL)	0.96 ± 0.22 (0.51–1.60)	1.07 ± 0.28 (0.67–1.90)	<0.001

Abbreviations: Data are presented as mean ± standard deviation (minimum–maximum). Independent samples *t*-test was used for comparisons. WBC, white blood cell; CRP, C-reactive protein; NLR, neutrophil-to-lymphocyte ratio; LMR, lymphocyte-to-monocyte ratio; PLR, platelet-to-lymphocyte ratio; PNI, prognostic nutritional index; HbA1c, hemoglobin A1c. Bold values indicate statistically significant differences between groups.

**Table 5:** Prognostic Performance of Different Scores in Predicting Two-Year Mortality.

Index	AUC (95% CI)	*p*-Value	Optimal Cut-Off	Sensitivity (%)	Specificity (%)	+LR	−LR	NPV (%)
CALLY	0.698 (0.628–0.768)	<0.001	≤1.11	71.7	67.9	2.23	0.42	90.7
GNRI	0.618 (0.529–0.707)	0.009	≤99.90	60.0	74.1	2.31	0.54	88.2
PNI	0.618 (0.541–0.694)	0.003	≤56.85	70.0	55.6	1.58	0.54	88.2
NLR	0.585 (0.503–0.666)	0.043	≥3.41	50.0	68.7	1.60	0.73	84.8
LMR	0.572 (0.488–0.656)	0.091	≤2.18	43.3	72.4	1.57	0.78	83.8
Albumin	0.561 (0.475–0.647)	0.162	≤4.05	60.0	55.6	1.35	0.72	84.9
NPSSCORE	0.559 (0.484–0.634)	0.122	≥1.5	81.7	32.1	1.20	0.57	87.6
SII	0.516 (0.436–0.596)	0.694	≤797.5	71.7	36.6	1.13	0.77	84.0
PLR	0.476 (0.394–0.557)	0.559	≥135.9	53.3	51.9	1.11	0.90	81.8

Abbreviations: AUC, Area Under the Curve; CI, Confidence Interval; CALLY, CRP–Albumin–Lymphocyte Index; PNI, Prognostic Nutritional Index; NLR, Neutrophil-to-Lymphocyte Ratio; LMR, Lymphocyte-to-Monocyte Ratio; SII, Systemic Inflammatory Index; GNRI, Geriatric Nutritional Risk Index; PLR, Platelet-to-Lymphocyte Ratio; +LR, Positive Likelihood Ratio; −LR, Negative Likelihood Ratio; NPV, Negative Predictive Value.

**Table 6:** Prognostic Factors for All-Cause Mortality Based on Univariate and Multivariate Cox Models.

	HR [95% CI]	*p* Value	HR [95% CI]	*p* Value
Age	1.03 [0.98, 1.07]	0.227	1.01 [0.97, 1.06]	0.655
Sex (Male)	0.95 [0.57, 1.58]	0.843	1.24 [0.73, 2.10]	0.433
Low CALLY Index (High Risk) ^a^	4.59 [2.61, 8.05]	<0.001	3.80 [2.03, 7.11]	<0.001
Ejection Fraction	0.93 [0.91, 0.94]	<0.001	0.93 [0.91, 0.94]	<0.001
Atrial Fibrillation	2.76 [1.66, 4.59]	<0.001	-	-
Hypertension	2.62 [1.24, 5.51]	0.011	-	-
Diabetes Mellitus	2.80 [1.68, 4.67]	<0.001	2.19 [1.26, 3.81]	0.005
Chronic Kidney Disease	4.89 [2.90, 8.24]	<0.001	3.47 [2.01, 6.00]	<0.001
Smoking	2.21 [1.27, 3.84]	0.005	-	-
Coronary Artery Disease	1.62 [0.97, 2.71]	0.066	-	-
Anemia	1.51 [0.91, 2.50]	0.112	-	-

Abbreviations: HR, Hazard Ratio; CI, Confidence Interval; CALLY, CRP–Albumin–Lymphocyte Index. ^a^ The CALLY index is a composite score where a lower value indicates higher clinical risk.

## Data Availability

Data available on request due to restrictions (e.g., privacy, legal or ethical reasons).

## Supplementary Materials

The following supporting information can be downloaded at: https://www.mdpi.com/article/10.3390/medicina62040755/s1, Figure S1: Propensity score matching analysis based on age showing age distribution before and after matching and standardized mean difference reduction.

## References

[1] Généreux P., Sharma R.P., Cubeddu R.J., Aaron L., Abdelfattah O.M., Koulogiannis K.P., Marcoff L., Naguib M., Kapadia S.R., Makkar R.R. (2023). The Mortality Burden of Untreated Aortic Stenosis. J. Am. Coll. Cardiol..

[2] Smith C.R., Leon M.B., Mack M.J., Miller D.C., Moses J.W., Svensson L.G., Tuzcu E.M., Webb J.G., Fontana G.P., Makkar R.R. (2011). Transcatheter versus Surgical Aortic-Valve Replacement in High-Risk Patients. N. Engl. J. Med..

[3] Hecht S., Giuliani C., Nuche J., Farjat Pasos J.I., Bernard J., Tastet L., Abu-Alhayja’a R., Beaudoin J., Côté N., DeLarochellière R. (2024). Multimarker Approach to Improve Risk Stratification of Patients Undergoing Transcatheter Aortic Valve Implantation. J. Am. Coll. Cardiol. Adv..

[4] Chen S.-Y., Kong X.-Q., Zhang J.-J. (2024). Pathological Mechanism and Treatment of Calcified Aortic Stenosis. Cardiol. Rev..

[5] Wernio E., Małgorzewicz S., Dardzińska J.A., Jagielak D., Rogowski J., Gruszecka A., Klapkowski A., Bramlage P. (2019). Association between Nutritional Status and Mortality after Aortic Valve Replacement Procedure in Elderly with Severe Aortic Stenosis. Nutrients.

[6] Ji H., Luo Z., Ye L., He Y., Hao M., Yang Y., Tao X., Tong G., Zhou L. (2024). Prognostic Significance of C-Reactive Protein-Albumin-Lymphocyte (CALLY) Index after Primary Percutaneous Coronary Intervention in Patients with ST-Segment Elevation Myocardial Infarction. Int. Immunopharmacol..

[7] Güven B., Deniz M.F., Geylan N.A., Kültürsay B., Dönmez A., Bulat Z., Gül Ö.B., Kaya M., Oktay V. (2025). A Novel Indicator of All-Cause Mortality in Acute Coronary Syndrome: The CALLY Index. Biomark. Med..

[8] Akdoğan O., Yücel K.B., Yazıcı O., Özet A., Özdemir N. (2025). Assessment of the CALLY Index, a Novel Immunonutrivite Marker, in Perioperatively Treated Gastric Cancer Patients: Prognostic Value of the CALLY Index in Gastric Cancer. Gazi Med. J..

[9] Yang M., Lin S.-Q., Liu X.-Y., Tang M., Hu C.-L., Wang Z.-W., Zhang Q., Zhang X., Song M.-M., Ruan G.-T. (2023). Association between C-Reactive Protein-Albumin-Lymphocyte (CALLY) Index and Overall Survival in Patients with Colorectal Cancer: From the Investigation on Nutrition Status and Clinical Outcome of Common Cancers Study. Front. Immunol..

[10] Zhu D., Lin Y.-D., Yao Y.-Z., Qi X.-J., Qian K., Lin L.-Z. (2024). Negative Association of C-Reactive Protein-Albumin-Lymphocyte Index (CALLY Index) with All-Cause and Cause-Specific Mortality in Patients with Cancer: Results from NHANES 1999–2018. BMC Cancer.

[11] Kucukosmanoglu M., Kilic S., Urgun O.D., Sahin S., Yildirim A., Sen O., Kurt İ.H. (2021). Impact of Objective Nutritional Indexes on 1-Year Mortality after Transcatheter Aortic Valve Implantation: A Prospective Observational Cohort Study. Acta Cardiol..

[12] Gitmez M., Güzel T., Kis M., Coskun F., İsik M.A., Aktan A., Kilic R., Demir M., Ertas F. (2025). The Performance of the NAPLES Prognostic Score in Predicting One-Year Mortality and Major Adverse Cardiovascular Events after Transcatheter Aortic Valve Implantation in Patients with Severe Aortic Stenosis. Pol. Heart J. (Kardiol. Pol.).

[13] Tosu A.R., Kalyoncuoglu M., Biter H.İ., Cakal S., Selcuk M., Çinar T., Belen E., Can M.M. (2021). Prognostic Value of Systemic Immune-Inflammation Index for Major Adverse Cardiac Events and Mortality in Severe Aortic Stenosis Patients after TAVI. Medicina.

[14] Panç C., Yılmaz E., Gürbak İ., Uzun F., Ertürk M. (2020). Effect of Prognostic Nutritional Index on Short-Term Survival after Transcatheter Aortic Valve Implantation. Arch. Turk. Soc. Cardiol..

[15] Shibata K., Yamamoto M., Kano S., Koyama Y., Shimura T., Kagase A., Yamada S., Kobayashi T., Tada N., Naganuma T. (2018). Importance of Geriatric Nutritional Risk Index Assessment in Patients Undergoing Transcatheter Aortic Valve Replacement. Am. Heart J..

[16] Birinci Ş. (2023). A Digital Opportunity for Patients to Manage Their Health: Turkey National Personal Health Record System (The e-Nabız). Balk. Med. J..

[17] Généreux P., Piazza N., Alu M.C., Nazif T., Hahn R.T., Pibarot P., Bax J.J., Leipsic J.A., Blanke P., VARC-3 Writing Committee (2021). Valve Academic Research Consortium 3: Updated Endpoint Definitions for Aortic Valve Clinical Research. J. Am. Coll. Cardiol..

[18] Qin P., Ho F.K., Celis-Morales C.A., Pell J.P. (2025). Association between Systemic Inflammation Biomarkers and Incident Cardiovascular Disease in 423,701 Individuals: Evidence from the UK Biobank Cohort. Cardiovasc. Diabetol..

[19] Desai M.Y., Braunwald E. (2025). The Pathophysiologic Basis and Management of Calcific Aortic Valve Stenosis: JACC State-of-the-Art Review. J. Am. Coll. Cardiol..

[20] Sinning J.-M., Scheer A.-C., Adenauer V., Ghanem A., Hammerstingl C., Schueler R., Müller C., Vasa-Nicotera M., Grube E., Nickenig G. (2012). Systemic Inflammatory Response Syndrome Predicts Increased Mortality in Patients after Transcatheter Aortic Valve Implantation. Eur. Heart J..

[21] Özbek M., Acun B., Arık B., Demir M., Oylumlu M., Toprak N. (2022). Prognostic Value of Nutritional and Inflammatory Scores in Transcatheter Aortic Valve Replacement Patients. Dicle Med. J..

[22] Koseki K., Yoon S.-H., Kaewkes D., Koren O., Patel V., Kim I., Sharma R., Sekhon N., Chakravarty T., Nakamura M. (2021). Impact of the Geriatric Nutritional Risk Index in Patients Undergoing Transcatheter Aortic Valve Implantation. Am. J. Cardiol..

[23] Rawat A., Goyal P., Ahsan S.A., Surya Srivyshnavi K.S., Hannan Asghar A., Riyalat A.A., Wei C.R., Khan A. (2025). The Prognostic Value of Neutrophil-to-Lymphocyte Ratio on Mortality in Patients Undergoing Transcatheter Aortic Valve Implantation: A Systematic Review and Meta-Analysis. Cureus.

[24] Trimarchi G., Pizzino F., Lilli A., De Caterina A.R., Esposito A., Dalmiani S., Mazzone A., Di Bella G., Berti S., Paradossi U. (2024). Advanced Lung Cancer Inflammation Index as Predictor of All-Cause Mortality in ST-Elevation Myocardial Infarction Patients Undergoing Primary Percutaneous Coronary Intervention. J. Clin. Med..

[25] Sousa A.L.S., Carvalho L.A.F., Salgado C.G., de Oliveira R.L., e Lima L.C.C.L., de Mattos N.D.F.G., Fagundes F.E.S., Colafranceschi A.S., Mesquita E.T. (2021). C-Reactive Protein as a Prognostic Marker of 1-Year Mortality after Transcatheter Aortic Valve Implantation in Aortic Stenosis. Arq. Bras. Cardiol..

[26] Bogdan A., Barbash I.M., Segev A., Fefer P., Bogdan S.N., Asher E., Fink N., Hamdan A., Spiegelstein D., Raanani E. (2016). Albumin Correlates with All-Cause Mortality in Elderly Patients Undergoing Transcatheter Aortic Valve Implantation. EuroIntervention.

[27] Yamamoto M., Shimura T., Kano S., Kagase A., Kodama A., Sago M., Tsunaki T., Koyama Y., Tada N., Yamanaka F. (2017). Prognostic Value of Hypoalbuminemia After Transcatheter Aortic Valve Implantation (from the Japanese Multicenter OCEAN-TAVI Registry). Am. J. Cardiol..

[28] Al-Kindi S.G., Attizzani G.F., Decicco A.E., Alkhalil A., Nmai C., Longenecker C.T., Parikh S., Lederman M.M., Dalton J., Zidar D.A. (2018). Lymphocyte Counts Are Dynamic and Associated with Survival after Transcatheter Aortic Valve Replacement. Struct. Heart.

