# Supplementary material for: Prognostic Value of the CALLY Index in Predicting All-Cause Mortality After Transcatheter Aortic Valve Implantation: A Two-Year Follow-Up Study
Source: Medicina (Kaunas). 2026 Apr 15;62(4):755. doi: 10.3390/medicina62040755 (PMC13117720; doi:10.3390/medicina62040755)
Supplement: Supplementary file 1 [file medicina-62-00755-s001.zip › medicina-4184472-supplementary.pdf]

Supplementary Figure S1

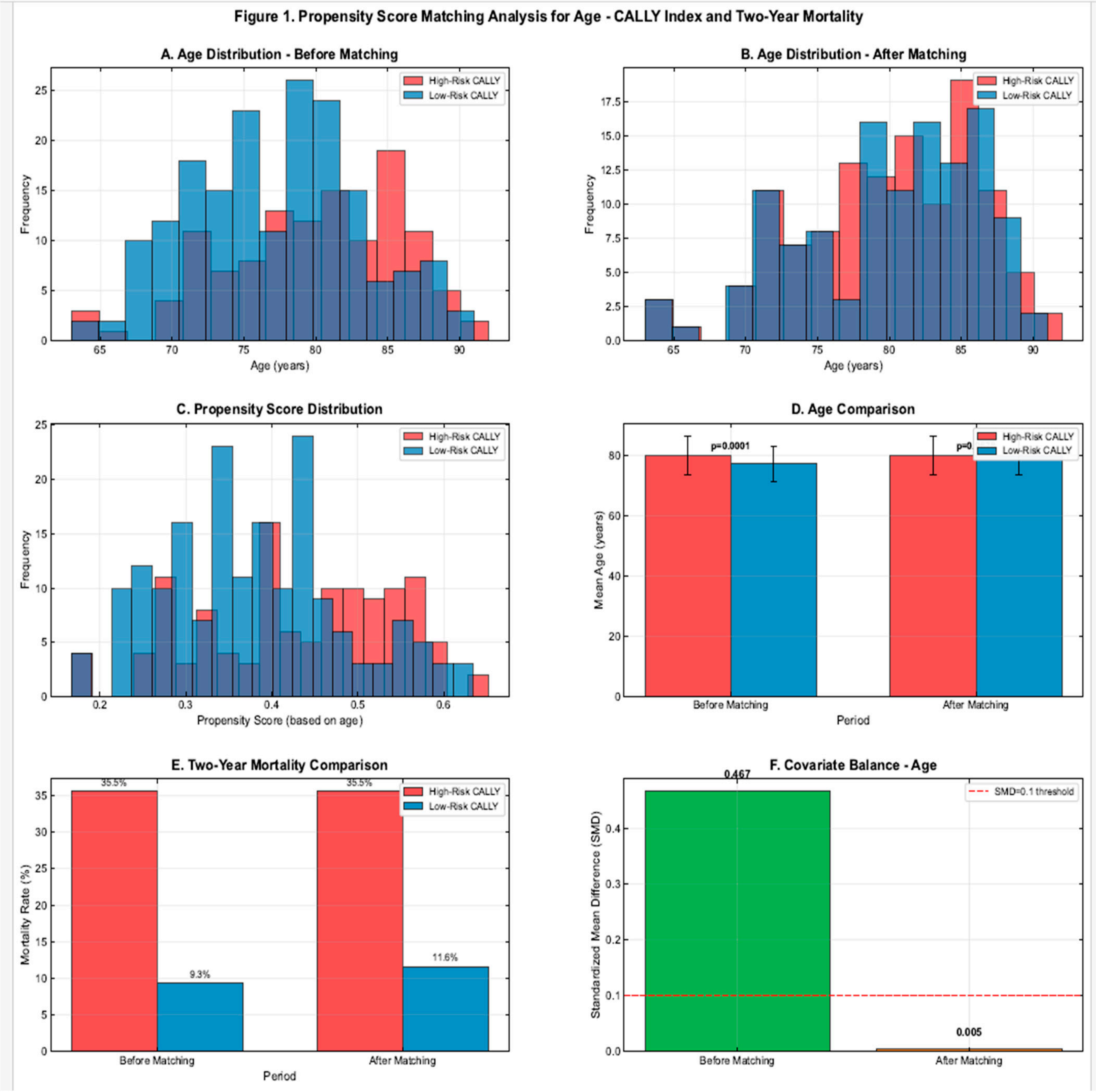

Propensity score matching analysis based on age showing age distribution before and after matching and standardized mean difference reduction.
